# Heme oxygenase 1 governs the cytoskeleton at filopodia: pulling the brakes on the migratory capacity of prostate tumoral cells

**DOI:** 10.1038/cddiscovery.2017.20

**Published:** 2017-04-03

**Authors:** Alejandra Paez, Elba Vazquez, Geraldine Gueron

**Affiliations:** 1 Department of Biological Chemistry, FCEN, University of Buenos Aires, IQUIBICEN-CONICET, Buenos Aires, Argentina

This paper refers to Paez *et al.*^1^ Prostate cancer (PCa) is the second leading cause of cancer death in men in the United States.^2^ Incidence increases with patient age and represents the most important risk factor. Localized PCa can be cured in most cases, but when the disease escapes the confines of the gland, the prospects for cure decrease drastically. Androgen ablation is the most effective way of halting PCa progression, but given sufficient time, growth of the cancer resumes in most cases and the disease becomes castration resistant (castration-resistant PCa (CRPC)).^3^ No therapy is curative for patients with CRPC, thus emerging a critical need to identify new therapy targets. Advanced PCa has been associated to the loss of cell adhesion molecules at adherens junctions.^4^ The delicate equilibrium between the cell pushing and pulling forces drive leading edge dynamics and cell migration. Interdigitating filopodia are vital for the proper alignment and establishment of the initial cell–cell adhesions, known as adhesion zippering, an event that contributes significantly to reduce the metastatic phenotype of tumor cells.^5^

Heme oxygenase 1 (HO-1) is as a stress response protein and a critical mediator of cellular homeostasis.^6^ HO-1 is also implicated in the modulation of cellular adhesion in PCa, upregulating E-cadherin and *β*-catenin expression, and relocating them to the cell membrane,^7^ favoring a more epithelial phenotype. However, it is yet unclear which are the HO-1 interactors and how they cooperate in the regulation of the cytoskeleton organization. Paez *et al.*^1^ undertook an in-depth mass spectrometry-based proteomics study to build the HO-1 interactome in PCa. Fifty-six HO-1 differentially associated proteins were identified including a subset of proteins responsible for the regulation of the cytoskeleton dynamics with potential clinical relevance in PCa, such as Heat shock 27 kDa protein (HSPB1), Gelsolin (GSN), LIM and SH3 Protein 1 (LASP1) protein, Muskelin (MKLN1) and Tropomodulin 3 (TMOD3). It was also demonstrated that HO-1 modulation impacts directly in the morphology of PCa cells, modulating the actin stress fibers, cell protrusions, cell contacts and cell migration, reflecting a less invasive and less motile phenotype (Figure 1).

Further, through a multi ‘omics’ approach, new aspects regarding the mechanistic strategy that HO-1 uses to alter protrusive forces and adhesive behavior of tumor cells were identified. Authors integrated RNA-seq data of PCa cells overexpressing HO-1 both genetically and pharmacologically, together with the HO-1 interactome unveiling significant alterations of four molecular pathways related to cell adhesion and cell–cell communication: ANXA2/HMGA1/POU3F1; NFRSF13/GSN; TMOD3/RAI14/VWF; PLAT/PLAU. Of note, HO-1 downmodulates the uPA/uPAR directly impacting on Rho GTPases through the alpha V-Beta 3 integrin receptor, which in turn affects filopodia formation. HO-1 also binds Gelsolin, STAT3 and HSPB1, potentially supporting its implication in filopodia regulation and gives ground to HO-1 involvement at the molecular level in the modulation of the cytoskeleton pathways.

Overall, this work breaks down two paradigms: first, filopodia structures increase the potentiality of tumor cells to adhere to each other rather than to increase the migratory and invasive potential of cells, demonstrating that the augmented number of filopodia per cell is not associated with a more motile phenotype; second, HO-1 actively participates in the regulation of actin dynamics at cellular protrusions rather than to be confined to its microsomal function (heme degradation). Further, new aspects of the mechanistic strategy by which HO-1 alters protrusive forces and the adhesive behavior of tumor cells are revealed, showcasing its relevance as a key homeostatic factor against PCa.

## Conclusion

We propose that HO-1 and its interactors reprogram PCa cells and modify the tumoral microenvironment, favoring a less aggressive phenotype.

## Figures and Tables

**Figure 1:**
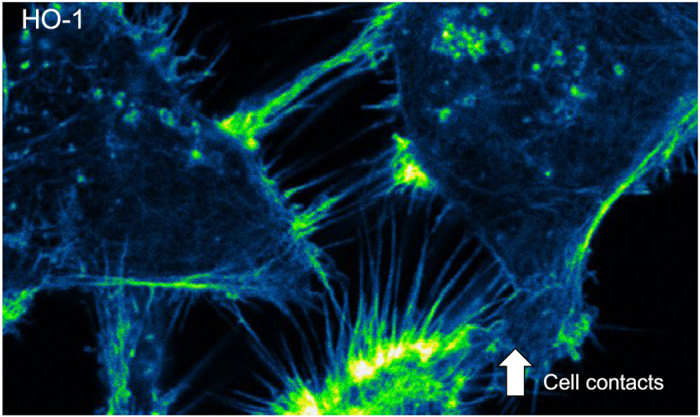
HO-1 overexpression in prostate cancer cells favors cell–cell contact, increasing filopodia zippering at the leading edge of cells. Prostate cancer cells overexpressing HO-1 were fixed, stained with rhodamine phalloidin (high affinity for F-actin) and imaged by confocal microscopy. The regions in which cell filopodia contacted two neighboring cells were divided into segments where the distance between the cells remained constant. An intensity profile for each of these sectors was determined using a custom-made algorithm to count contacts.
